# Real-time intravascular photoacoustic-ultrasound imaging of lipid-laden plaque in human coronary artery at 16 frames per second

**DOI:** 10.1038/s41598-017-01649-9

**Published:** 2017-05-03

**Authors:** Jie Hui, Yingchun Cao, Yi Zhang, Ayeeshik Kole, Pu Wang, Guangli Yu, Gregory Eakins, Michael Sturek, Weibiao Chen, Ji-Xin Cheng

**Affiliations:** 1 Department of Physics and Astronomy, Purdue University, West Lafayette, IN 47907 USA; 2 Weldon School of Biomedical Engineering, Purdue University, West Lafayette, IN 47907 USA; 3 Department of Cellular and Integrative Physiology, Indiana University School of Medicine, Indianapolis, IN 46202 USA; 4 Nanjing Institute of Advanced Laser Technology, Nanjing, 210038 China; 5 Jonathan Amy Facility for Chemical Instrumentation, Purdue University, West Lafayette, IN 47907 USA; 6 Shanghai Institute of Optics and Fine Mechanics, Chinese Academy of Sciences, Shanghai, 201800 China; 7 Department of Chemistry, Purdue University, West Lafayette, IN 47907 USA; 8 Purdue Institute for Inflammation, Immunology, and Infectious Disease, Purdue University, West Lafayette, IN 47907 USA

## Abstract

Intravascular photoacoustic-ultrasound (IVPA-US) imaging is an emerging hybrid modality for the detection of lipid-laden plaques, as it provides simultaneous morphological and lipid-specific chemical information of an artery wall. Real-time imaging and display at video-rate speed are critical for clinical utility of the IVPA-US imaging technology. Here, we demonstrate a portable IVPA-US system capable of imaging at up to 25 frames per second in real-time display mode. This unprecedented imaging speed was achieved by concurrent innovations in excitation laser source, rotary joint assembly, 1 mm IVPA-US catheter size, differentiated A-line strategy, and real-time image processing and display algorithms. Spatial resolution, chemical specificity, and capability for imaging highly dynamic objects were evaluated by phantoms to characterize system performance. An imaging speed of 16 frames per second was determined to be adequate to suppress motion artifacts from cardiac pulsation for *in vivo* applications. The translational capability of this system for the detection of lipid-laden plaques was validated by *ex vivo* imaging of an atherosclerotic human coronary artery at 16 frames per second, which showed strong correlation to gold-standard histopathology. Thus, this high-speed IVPA-US imaging system presents significant advances in the translational intravascular and other endoscopic applications.

## Introduction

Coronary artery disease remains the leading cause of morbidity and mortality throughout the world. Atherosclerosis, a major form of coronary artery disease, occurs in many different forms and can be distinguished by morphologic classification into different plaque types^1^. Among them, the thin-capped fibroatheroma has been understood to be the most “vulnerable” plaque type, as evidence shows it is the most prone to rupture and progress to thrombosis and acute coronary syndrome^1–4^. Thin-capped fibroatheromas are grossly defined by hallmarks of a large lipid-rich necrotic core, a thin fibrous cap, and inflammatory infiltrate^2, 4^. In addition, these plaques are often structurally non-obstructive to moderately obstructive, thus clinically unidentifiable by routine angiography and stress testing^2, 5, 6^. Currently, there are no clinically available imaging tools to reliably and accurately detect vulnerable plaques^7–9^. Angiographic techniques, including X-ray angiography, computed tomography angiography, and magnetic resonance angiography, are limited to visualizing areas of severe luminal narrowing. Intravascular ultrasound (IVUS) provides the overall morphology of artery wall for quantification of the plaque burden and monitoring disease progression, but lacks chemical selectivity to identify plaque composition. Virtual-histology IVUS, based on radiofrequency (RF) analysis of the ultrasound signal, provides valuable information of the plaque composition^10, 11^, but still has not been thoroughly validated^12, 13^. Intravascular near-infrared spectroscopy (NIRS) has been shown to reliably detect lipid-rich plaques in the artery wall^14, 15^, but lacks imperative depth resolution to quantify the size and location of the lipid deposition. Intravascular optical coherence tomography can accurately detect fibrous cap thickness with micron-scale resolution^16^, but lacks sufficient imaging depth and chemical selectivity to wholly determine plaque composition. These gaps, along with the increasing prevalence of coronary artery disease, highlight an unmet clinical need for a chemically-selective imaging modality with spatial resolution to advance the detection, understanding, and treatment of lipid-laden vulnerable plaques.

Dual-mode intravascular photoacoustic-ultrasound (IVPA-US) imaging is a promising approach to bridge the aforementioned gaps. In this hybrid modality, IVPA channel maps the lipid-specific chemical composition over the entire artery wall with ultrasonic spatial resolution. Based on conversion of optical absorption into ultrasound signal, photoacoustic (PA) imaging provides chemical selectivity and deep penetration depth^17–20^. Specifically, the lipid-specific contrast is generated endogenously from the first overtone absorption of C-H bonds at 1.7 µm wavelength^21, 22^ and the PA signal is one order of magnitude greater than that of water and connective tissues^22^. Furthermore, IVPA imaging works concurrently with IVUS imaging, which can provide simultaneous morphological information. Therefore, a co-registered IVPA-US image provides complementary information necessary for advanced and quantitative assessment of lipid-laden vulnerable plaques.

The IVPA-US imaging technology is currently under active investigation for the identification of various tissue components^21, 23, 24^, contrast mechanism^21, 22^, optical excitation sources^25–27^, IVPA-US catheter designs^27–31^, and *ex vivo* and preclinical validations^32–34^. However, these works are limited by the use of slow imaging speeds (up to 5 frames per second (fps)^27^) as well as lack of real-time image display, which are necessary components for future *in vivo* applications where imaging must be at a sufficient speed to avoid motion artifacts from cardiac pulsation. Motion artifacts can lead to inaccurate spatial mapping and quantification of lipid deposition, and subsequent misinterpretation of plaque type. Furthermore, a lack of real-time processing and display capability would prevent user feedback necessary to adjust imaging parameters and location.

In this work, we overcame these limitations by presenting a portable IVPA-US system capable of imaging in real-time, up to 25 fps. In this system, a master oscillator power amplifier (MOPA)-pumped optical parametric oscillator (OPO) with a wavelength of 1.7 µm and a repetition rate of 2 kHz was used as the optical excitation source. A compact fiber-optic rotary joint provided efficient and stable optical coupling with a scanning speed up to 30 revolutions per second. A 1 mm diameter collinear IVPA-US catheter was built for high-sensitivity imaging. A differentiated A-line strategy, where the triggering frequency for US was doubled to that of PA, was applied to maximize IVPA-US imaging functionality. Lastly, LabView-based algorithms were developed to process and display IVPA-US images in real-time. These developments enabled the 25 fps imaging speed, a 5 times improvement over any previously reported IVPA-US imaging speed and comparable to the imaging speeds of current commercial IVUS and NIRS-IVUS systems^35^.

## Results

### High-speed real-time IVPA-US imaging system

**Figure 1:**
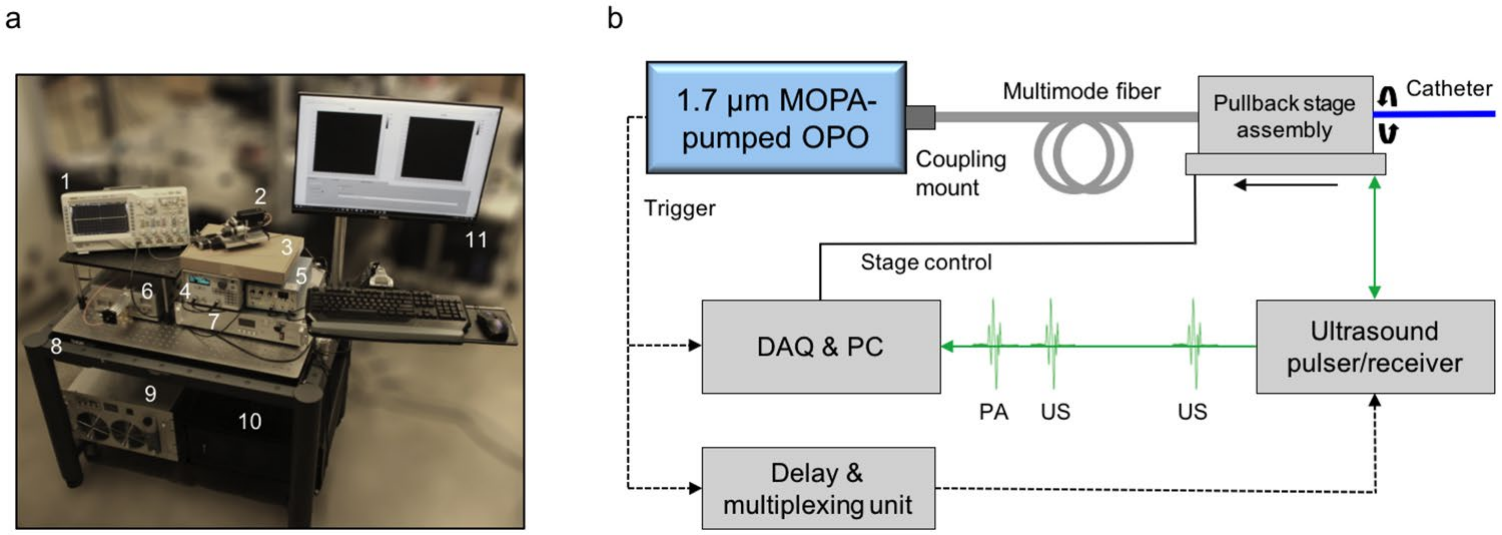
High-speed real-time IVPA-US imaging system. (**a**) Image of the portable IVPA-US imaging system. Major components: 1, oscilloscope; 2, fiber-optic rotary joint assembly; 3, IVPA-US catheter; 4, delay generator; 5, ultrasound pulser/receiver; 6, MOPA-pumped OPO; 7, laser controller; 8, mobile cart; 9, laser chiller; 10, PC and DAQ card; 11, image display monitor. (**b**) Schematic of IVPA-US imaging system layout. Dashed black line: trigger signal; solid black line: stage control cable; solid green line: radiofrequency signal.

We designed and developed the compact and portable IVPA-US imaging system as shown in Fig. 1(a). The detailed connections and controls of these components were shown in Fig. 1(b). Briefly, in the system, a 1.7 µm, 2 kHz MOPA-pumped OPO was used for high-speed optical excitation. Its output pulses were coupled to a multimode fiber through a coupling mount, then a fiber-optic rotary joint, and lastly the IVPA-US catheter tip. Initial ultrasound pulses generated from an ultrasound pulser were sent to the catheter tip through a slip ring. The delays between the ultrasound and optical pulses were precisely controlled by a delay generator. The B-scan and linear pullback of the catheter was conducted with the fiber-optic rotary joint assembly and rotation and pullback rate controlled by a computer. The sequentially generated PA and US signals were detected by the IVPA-US catheter, transmitted by the slip ring, amplified by the ultrasound receiver, digitized and recorded by a data acquisition (DAQ) card, and further processed and displayed in real-time. This IVPA-US system was capable of imaging in real-time at speed up to 25 fps. The imaging speed was achieved by innovations in several key components, which are presented as below.

**Figure 2:**
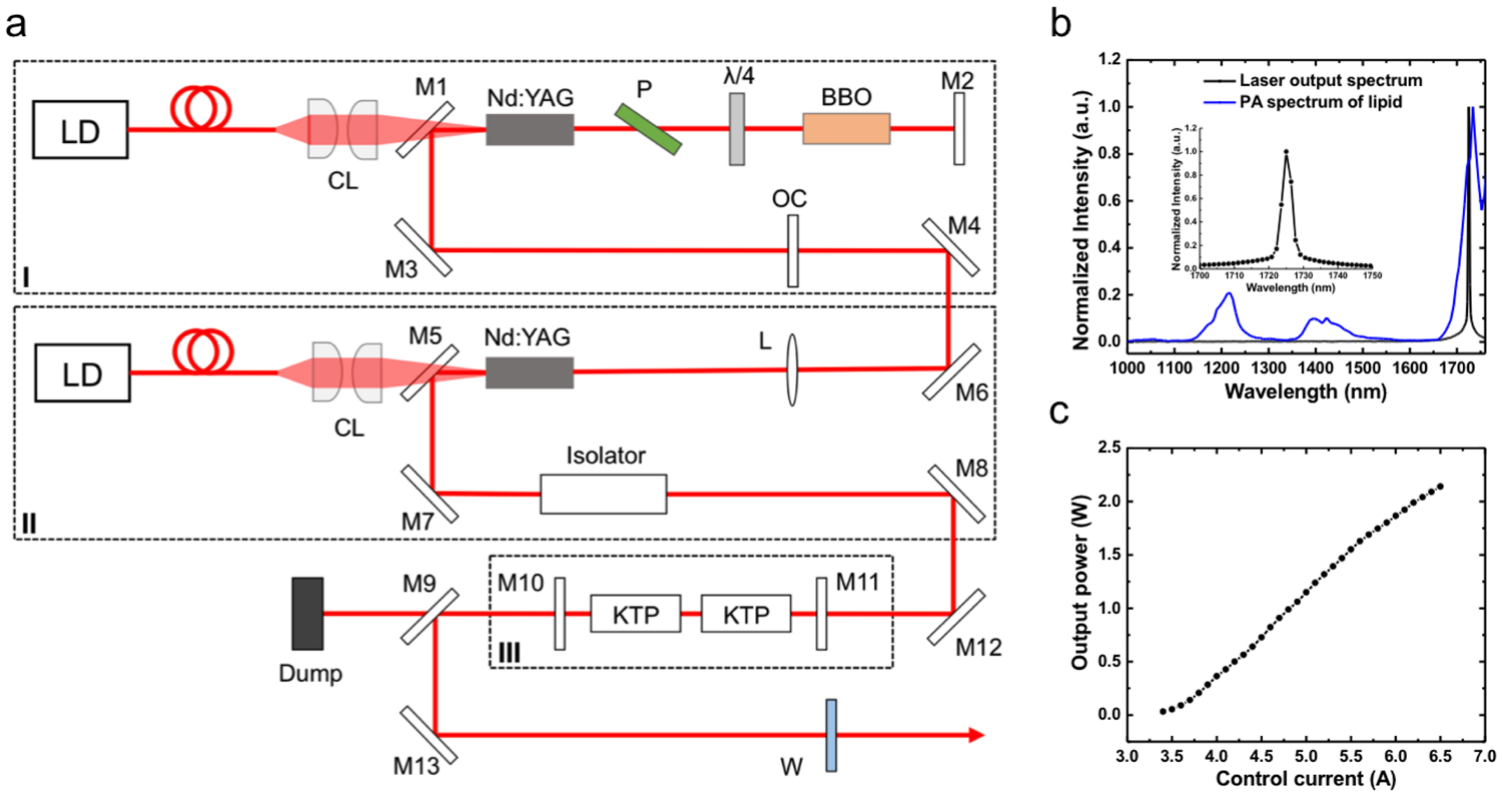
1.7 µm 2 kHz MOPA-pumped OPO for high-speed optical excitation. (**a**) Schematic of MOPA-pumped OPO. Dashed boxes labeled by I, II, and III highlight the master oscillator, optical power amplifier, and OPO, respectively. LD, laser diode; CL, coupling lens; M1 and M5, fold mirror; M2, M10, and M11, flat mirror; M3, M4, M6, M7, M8, M12, and M13, reflective mirror; M9, dichroic mirror; P, polarizer; BBO, beta barium borate Pockels cell; OC, output coupler; L, lens; KTP, potassium titanyl phosphate; W, output window. (**b**) Output wavelength of the laser (black curve), 1725 nm, matching the maximum peak in photoacoustic spectrum of lipids (blue curve). (**c**) Output power with control current. Other performance parameters can be found in Supplementary Fig. S1.

The determining factor for imaging speed is the laser pulse repetition rate, as each laser pulse corresponds to a depth-resolved A-line signal. In this system, a custom-built MOPA-pumped OPO was used for 2 kHz optical excitation, with the optical layout detailed in Fig. 2(a). The master oscillator provided 2.5 mJ and 15 ns laser pulses at 1064 nm with a 2 kHz repetition rate. The output was amplified to 6.0 mJ by a laser diode side-pumped optical power amplifier. Its wavelength was further shifted to 1.7 µm by a potassium titanyl phosphate (KTP)-based OPO. The performance of the laser output was further characterized. The final output wavelength was measured to be 1725 nm, which coincides with the first overtone transition frequency of C-H bonds (Fig. 2(b)). Thus, laser pulses at this wavelength were effectively absorbed by C-H bond-rich lipids, which generated strong PA signals. The output power was tunable in a range of 2.1 W, sufficient for lipid excitation (Fig. 2(c)). Other laser performance characterizations were shown in Supplementary Fig. S1. Collectively, this laser source was optimal for high-speed IVPA imaging of lipids.

**Figure 3:**
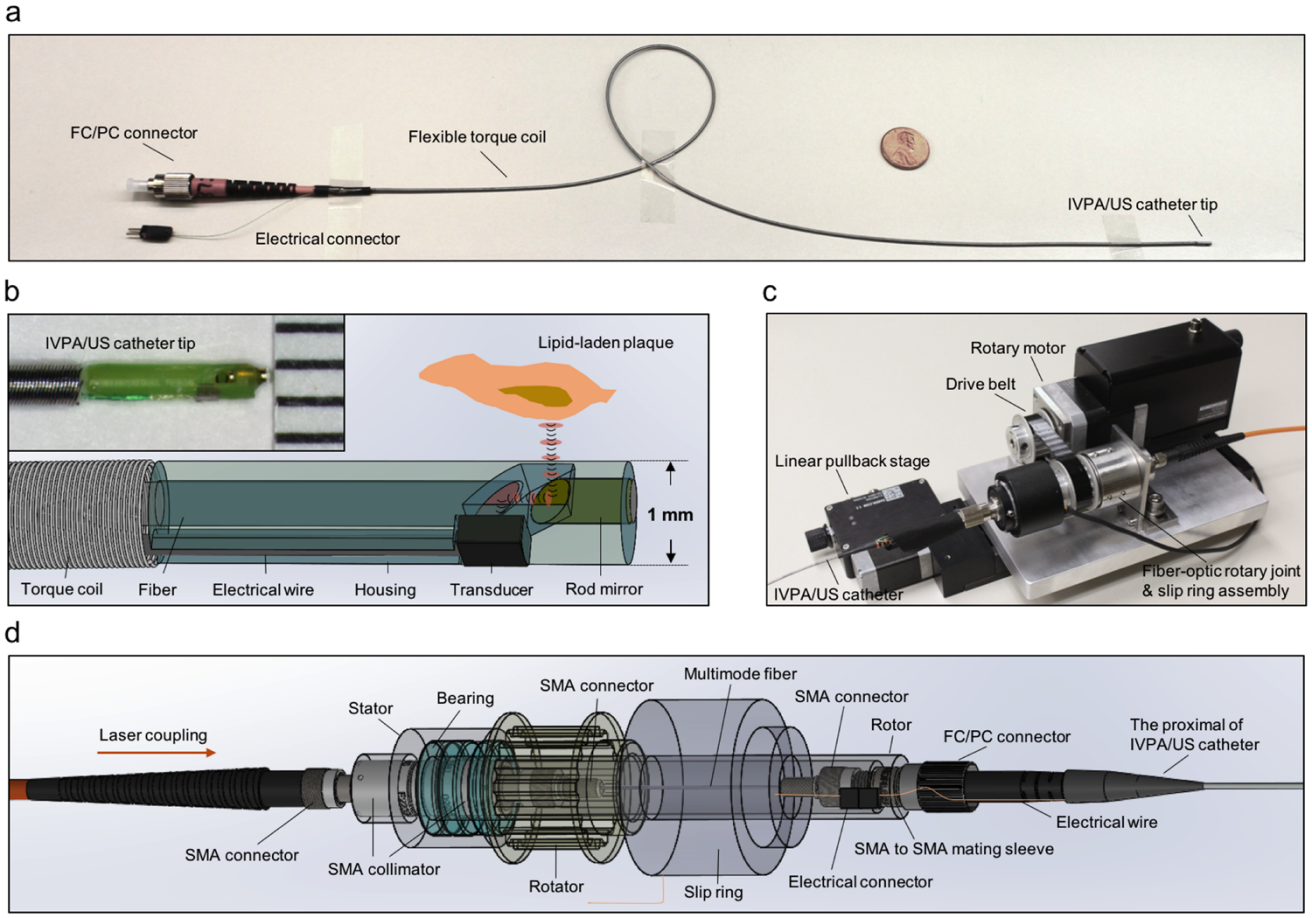
1-mm high-sensitivity catheter and fiber-optic rotary joint assembly for high-speed IVPA-US imaging. (**a**) Gross picture of flexible IVPA-US catheter with a 1 cent coin. Proximal end of the catheter was assembled with a FC/PC connector. (**b**) Schematic of 1-mm collinear IVPA-US catheter design. The red ellipses and black curves highlight the collinear paths of optical pulses and acoustic waves, respectively. Inset shows the actual picture of assembled catheter tip. Ruler scale: 1 mm. (**c**) Picture of overall fiber-optics rotary joint assembly. (**d**) Schematic of fiber-optic rotary joint for high-pulse-energy high-repetition-rate laser delivery.

The catheter used in the system had a collinear IVPA-US design with a diameter of 1 mm. Fig. 3(a) showed the actual image of a fully assembled collinear IVPA-US catheter, with the detailed schematic of the catheter imaging window shown in Fig. 3(b). In this design (further details found elsewhere^28^), the incident optical wave and its generated ultrasound wave were collinearly aligned, providing efficient overlap between the optical and acoustic paths over an imaging depth of >6 mm. In this work, we further miniaturized the catheter diameter to 1 mm, a clinically-compatible size adopted by most commercial IVUS systems. The reduction in diameter was achieved by aligning a miniaturized single element transducer (0.5 × 0.6 × 0.2 mm^3^), a multimode fiber with core diameter of 365 µm, and a reflection rod mirror with diameter of 365 µm in a 3-D printed housing. The beam path and beam characteristics were shown in Supplementary Fig. S2. The inset in Fig. 3(b) showed the actual image of an assembled IVPA-US catheter tip. This catheter provided high-sensitivity IVPA-US imaging capability and its size made access into human coronary artery samples feasible.

The fiber-optic rotary joint assembly is another key element that enabled the high-speed imaging. The assembly simultaneously provided optical coupling, RF signal transmission, fast rotary scan, and linear pullback. The compact and portable assembly was shown in Fig. 3(c). The rotary motor and the linear stage were used to provide rotary scan and linear pullback of catheter for 3-D imaging. The fiber-optic rotary joint was used to provide sufficient and stable optical coupling at high rotational speeds. The design of fiber-optic rotary joint was shown in Fig. 3(d). In this design, the use of two adjacent collimators and a mating sleeve enabled an overall coupling efficiency of 60% from the initial optical input to the final output at the catheter tip. Furthermore, the design significantly minimized the coupling efficiency variation usually caused by the mechanical rotation to 5.4%. Thus, the artery wall was uniformly excited in laser pulse energy at every angular position. The fiber-optic rotary joint was further driven by the motor with rotational speed up to 30 revolutions per second.

**Figure 4:**
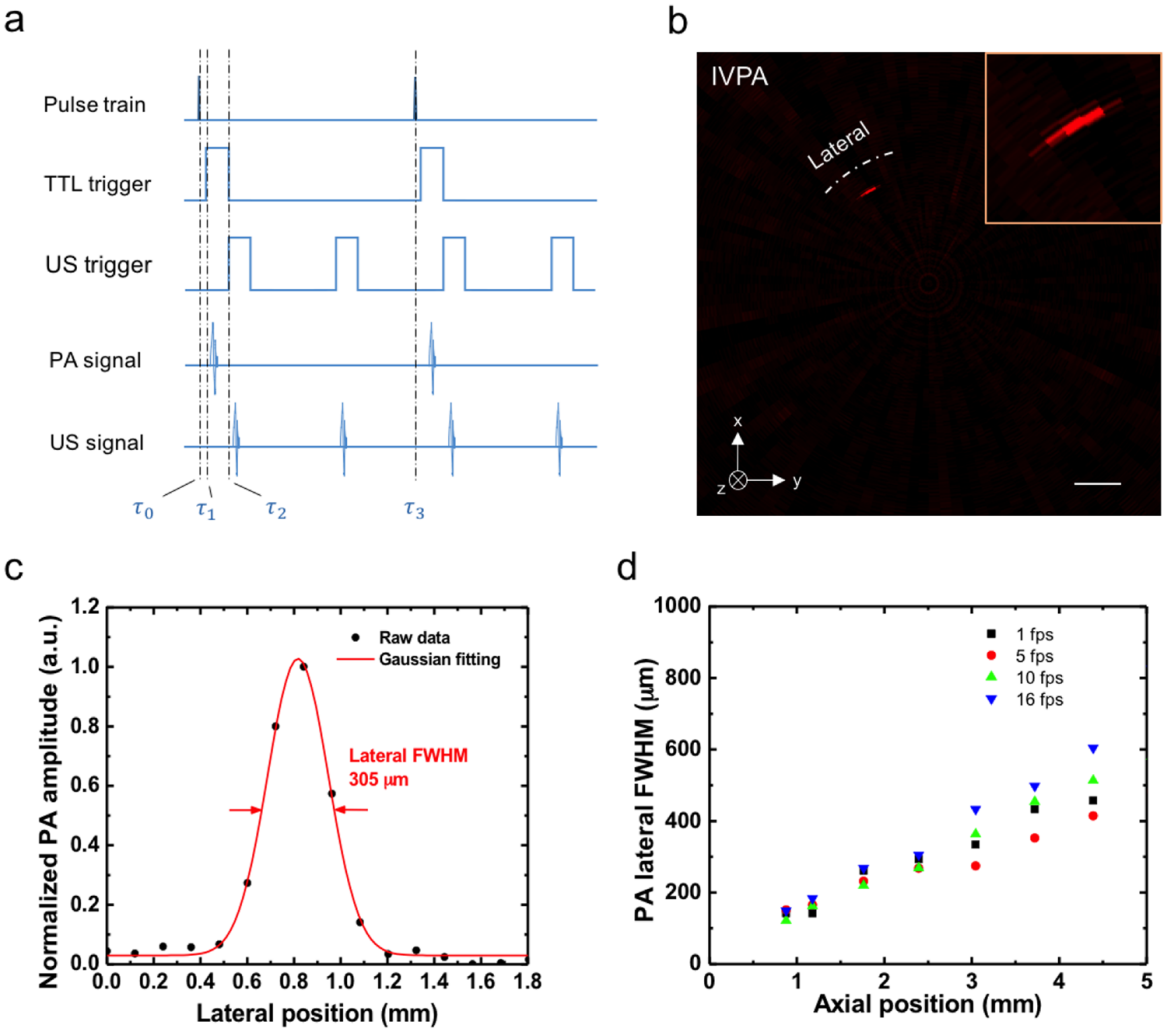
Differentiated A-line strategy and A-line number verification for IVPA-US imaging at 16 fps. (**a**) Timing diagram of IVPA-US imaging. ${\tau }_{0}$, 0 µs; ${\tau }_{1}$, 0.126 µs; ${\tau }_{2}$, 8.126 µs; ${\tau }_{3}$, 258.126 µs. TTL trigger refers to the trigger signal from laser. (**b**) Cross-sectional IVPA image of a single carbon fiber with diameter of 30 µm for spatial resolution characterization. The image shown was reconstructed by raw imaging data without A-line interpolation. Inset shows the zoom-in IVPA image. Scale bar: 1 mm. (**c**) Lateral plot of the carbon fiber in IVPA image at an axial position of 2.38 mm. The lateral resolution of 305 µm is estimated by the full with at half maximum (FWHM) of Gaussian fitted curve. (**d**) IVPA lateral resolution at different axial position at imaging speeds of 1, 5, 10, and 16 fps.

To maximize IVPA-US imaging functionality at high imaging speeds, a differentiated A-line strategy was designed. Fig. 4(a) showed the timing diagram for the A-line strategy. The transistor-transistor logic (TTL) signal from the laser was used to trigger DAQ card. The same trigger was delayed and frequency-doubled through multiplexing by a delay generator to trigger initial ultrasound pulses. As a result, the digitized signal in one acquisition cycle contained one PA signal segment and two US signal segments. Thus, the number of A-lines in a cross-sectional IVUS image was two times of that in IVPA image. Specifically, at 16 fps, 125 A-lines were used to reconstruct a cross-sectional IVPA image; 250 A-lines were used to reconstruct a cross-sectional IVUS image, comparable to current commercial systems. In order to verify the use of 125 A-lines in IVPA image, we characterized IVPA lateral resolution by imaging of a single 30 µm diameter carbon fiber submerged in heavy water. The cross-sectional IVPA image at 16 fps was shown in Fig. 4(b) and its lateral resolution of 305 µm was estimated by the full-width-at-half-maximum (FWHM) of the Gaussian fitted curve of the raw data points in the lateral direction at an axial position of 2.38 mm (Fig. 4(c)). At this axial position, there were 2.5 A-lines sampled in the lateral resolution of 305 µm, which indicates that signals in IVPA image were adequately sampled in the lateral direction based on the Nyquist sampling theorem (2.5 was calculated by L/(2 * pi * R/N), where L is the lateral resolution, N is the number of A-lines per cross-sectional image, and R is the axial position). Using the same methodology, there were constantly ~2.5 A-lines sampled in the lateral resolution at every axial position for 16 fps IVPA imaging (calculated based on the data in Fig. 4(d)). Thus, the use of 125 A-lines should provide adequate image quality for IVPA imaging. Meanwhile, we characterized the lateral resolutions at different axial positions at other imaging speeds (Fig. 4(d)). Notably, when the axial position was increased, the lateral resolution decreased, varying from 150 to 600 µm. However, there was no significant difference in lateral resolution at each axial position at different imaging speeds.

To achieve real-time display of IVPA-US images, we developed fast processing and display algorithms. The processing flow was shown in Supplementary Fig. S3 and the details were in Methods. Briefly, the digitized data containing non-meaningful signals was discarded in a preprocessing loop. The remaining PA and US segments were processed, reconstructed, and displayed in parallel IVPA and IVUS loops. A polar projection module was applied to convert IVPA and IVUS signals into polar images simultaneously. Through this program, we were able to image and display in real-time. (More details about the high-speed real-time IVPA-US imaging system can be found in Methods).

### IVPA-US imaging of pulsatile motion at different speeds

**Figure 5:**
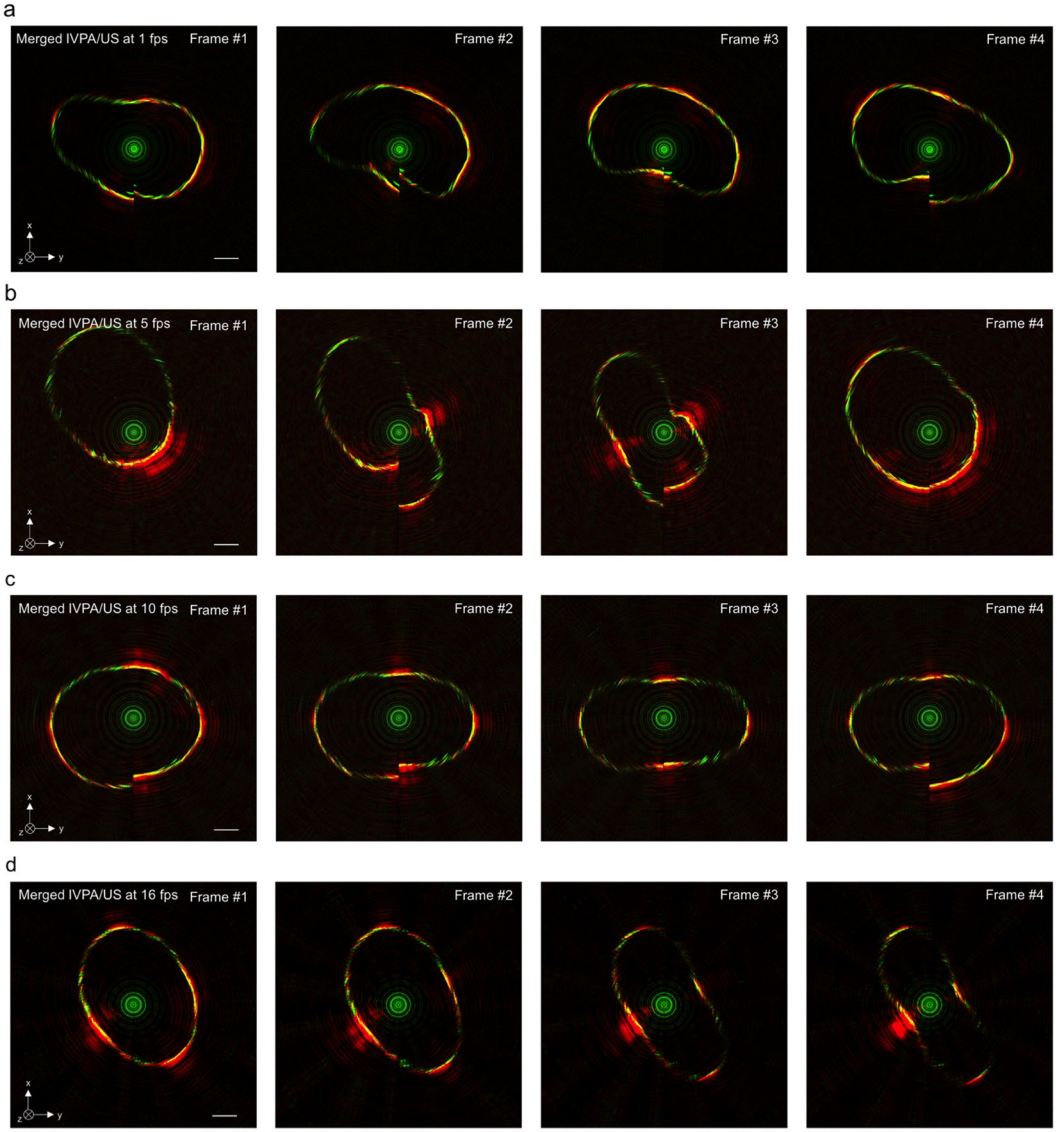
IVPA-US imaging of pulsatile motion at different speeds. Four consecutive IVPA-US frames selected at speed of (**a**) 1 fps, (**b**) 5 fps, (**c**) 10 fps, and (**d**) 16 fps highlighting the imaging distortion induced by pulsatile motion at lower imaging speed, while demonstrating the capability to suppress the motion artifacts at 16 fps. Complete comparison of IVPA-US imaging results at different imaging speed (1, 5, 10, 16, 20, and 25 fps) can be found in Supplementary Video S3. The depth field of view is 5.09 mm. Scale bar: 1 mm.

To find the optimal speed to image arteries with cardiac motion in *in vivo* settings, a phantom with pulsatile motion mimicking a human heartbeat was imaged at different speeds: 1, 5, 10, 16, 20, and 25 fps. Heat shrink tubes have a high absorption and can generate strong PA signal in a broad optical spectrum including 1.7 µm. Thus, we used a short heat shrink tube segment with a ~6 mm diameter in a heavy water environment as the phantom. At the time of imaging, the pulsatile motion was generated by mechanically pinching the tube at 1.2 Hz with forceps. The demonstration of real-time IVPA-US imaging of heat shrink tube at 16 fps and 25 fps can be found in Supplementary Video S1 and Supplementary Video S2, respectively. Supplementary Video S3 showed the merged IVPA-US images at speeds of 1, 5, 10, 16, 20, and 25 fps, respectively. In this video, IVPA and IVUS signals from the circumference of the phantom were detected at all imaging speeds. The imaging speed did not affect detection sensitivity and IVPA-US co-registration. However, IVPA-US imaging at speeds of 1 and 5 fps was not able to accurately capture 1.2 Hz pulsatile motion. The speed of 10 fps was acceptable, but its slow refresh rate was noticeable. However, there was no observable difference among the speeds of 16, 20, 25 fps in capturing the 1.2 Hz pulsatile motion. Thus, IVPA-US images at low imaging speeds were subject to distortion from motion artifacts. As contrasting examples, four consecutive IVPA-US images at 1 fps (Fig. 5(a)), 5 fps (Fig. 5(b)), 10 fps (Fig. 5(c)), and 16 fps (Fig. 5(d)) were used to demonstrate the distortion. At lower imaging speeds, there was obvious A-line signal mismatch at 6 o’clock, where the first and final A-lines in a cross-sectional image were located (e.g. Frames #2 and #4 in Fig. 5(a), Frames #2 and #3 in Fig. 5(b), Frame #4 in Fig. 5(c)). However, at 16 fps, the mismatch became negligible (Fig. 5(d)). Furthermore, the overall shape of shrink tube at lower speeds was distorted, especially for images at speeds of 1 and 5 fps (e.g. Frames #1—4 in Fig. 5(a), Frames #2 and #3 in Fig. 5(b)); while the distortion was successfully suppressed at 16 fps and its images reflected the real tube cross-sections (Fig. 5(d)). Lastly, at 16 fps, the dynamic change on tube shape from Frame #1 to #4 in Fig. 5(d) was continuous; but, at 1 fps, such continuity was not shown (Fig. 5(a)). Thus, a 16 fps imaging speed should be sufficient to avoid motion artifacts induced by cardiac pulsation for *in vivo* IVPA-US imaging.

### IVPA-US imaging of tissue phantom at 16 fps

**Figure 6:**
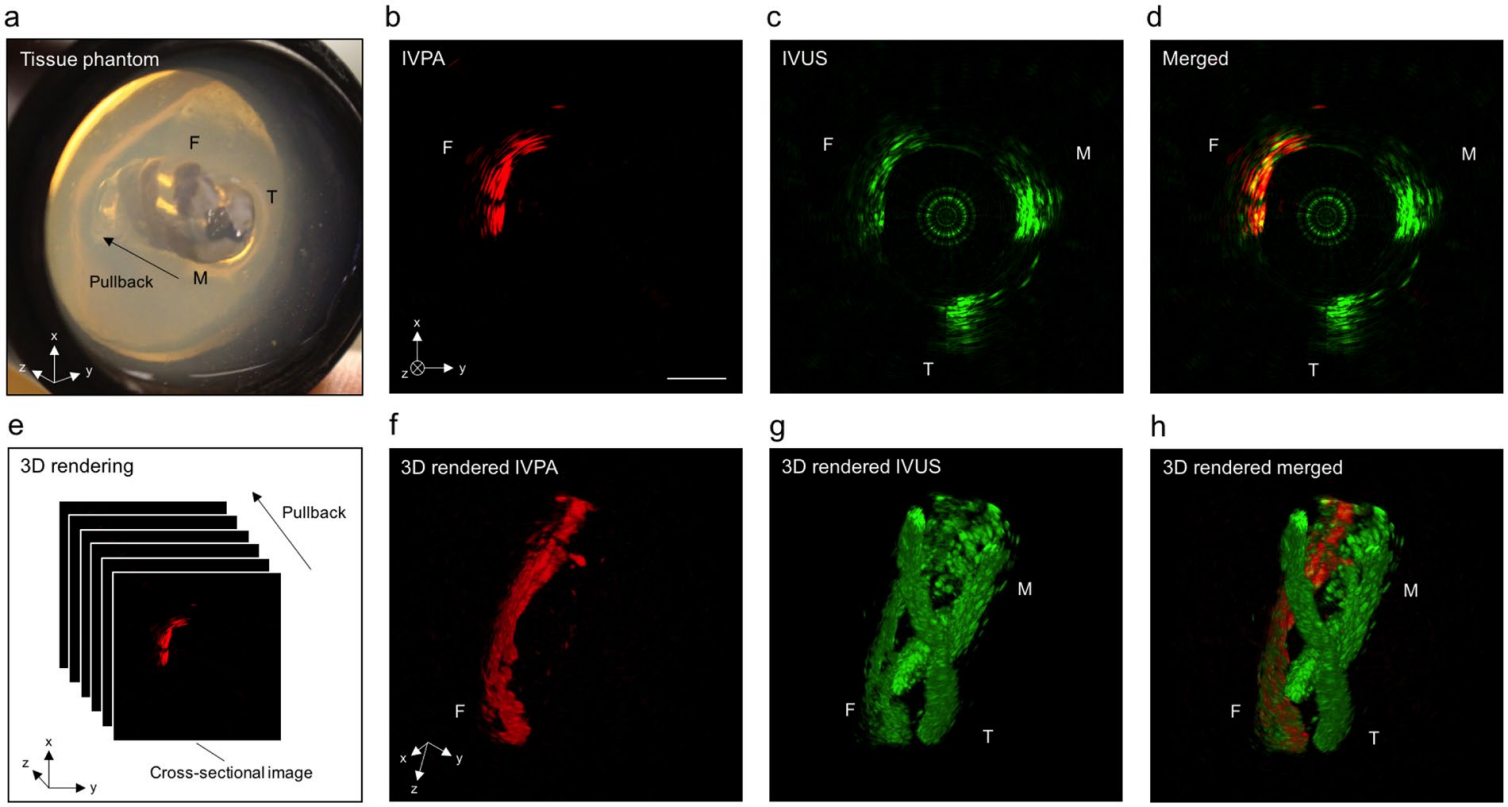
IVPA-US imaging of tissue phantom at 16 fps. (**a**) Picture of tissue phantom. F, intramuscular fat; T, tendon; M, muscle. Cross-sectional (**b**) IVPA, (**c**) IVUS, and (**d**) merged images of the tissue phantom. The scale bar and the coordinates in (**b**) is also applied to (**c**,**d**). (**e**) 3-D rendering method. 3-D rendered (**f**) IVPA, (**g**) IVUS, and (**h**) merged images of the tissue phantom with a pullback length of 10 mm. The coordinates in (**f**) is also applied to (**g**,**h**). Scale bar: 1 mm.

To test the chemical specificity of our imaging system, we imaged different tissue components, including intramuscular fat, tendon, and muscle tissue, at 16 fps. Intramuscular fat is rich in CH_2_ groups and has similar optical absorption at 1.7 µm as intravascular lipids, thus was used to mimic a lipid-laden plaque. Tendons and muscle tissue were used to mimic the medial layer of an artery, which is composed of vascular smooth muscle cells and collagen-rich extracellular matrix. These tissue components were harvested from a swine and dissected into small segments to be embedded into 2.5% agar gel as shown in Fig. 6(a). A central lumen was created in the phantom to insert the imaging catheter and the phantom was submerged in heavy water during the experiment. A 10 mm pullback of the phantom was captured at 16 fps with a pulse energy of 60 µJ (30.69 mJ/cm^2^ for laser fluence, calculation found in Supplementary Fig. S2) on the sample and a pullback speed of 0.5 mm/s. In the pullback, 320 frames of IVPA and IVUS data were acquired and reconstructed. Supplementary Video S4 showed the entire pullback. Fig. 6(b–d) showed the IVPA, IVUS, and merged images at one selected position, respectively. The image frames were 3-D rendered along the pullback direction (3-D rendering method was shown in Fig. 6(e)), showing the overall spatial distribution of each imaged tissue component (Fig. 6(f–h) and Supplementary Video S5). In the IVPA channel, only intramuscular fat generated signal, with a signal-to-noise ratio of 46.8 (Fig. 6(f)). However, in the IVUS channel, all three tissue components generated similar levels of signal. Thus, these results validated the chemical selectivity of our imaging system, indicating that lipid deposition in an artery can be specifically mapped.

### IVPA-US imaging of human coronary atherosclerosis and comparison to histopathology

**Figure 7:**
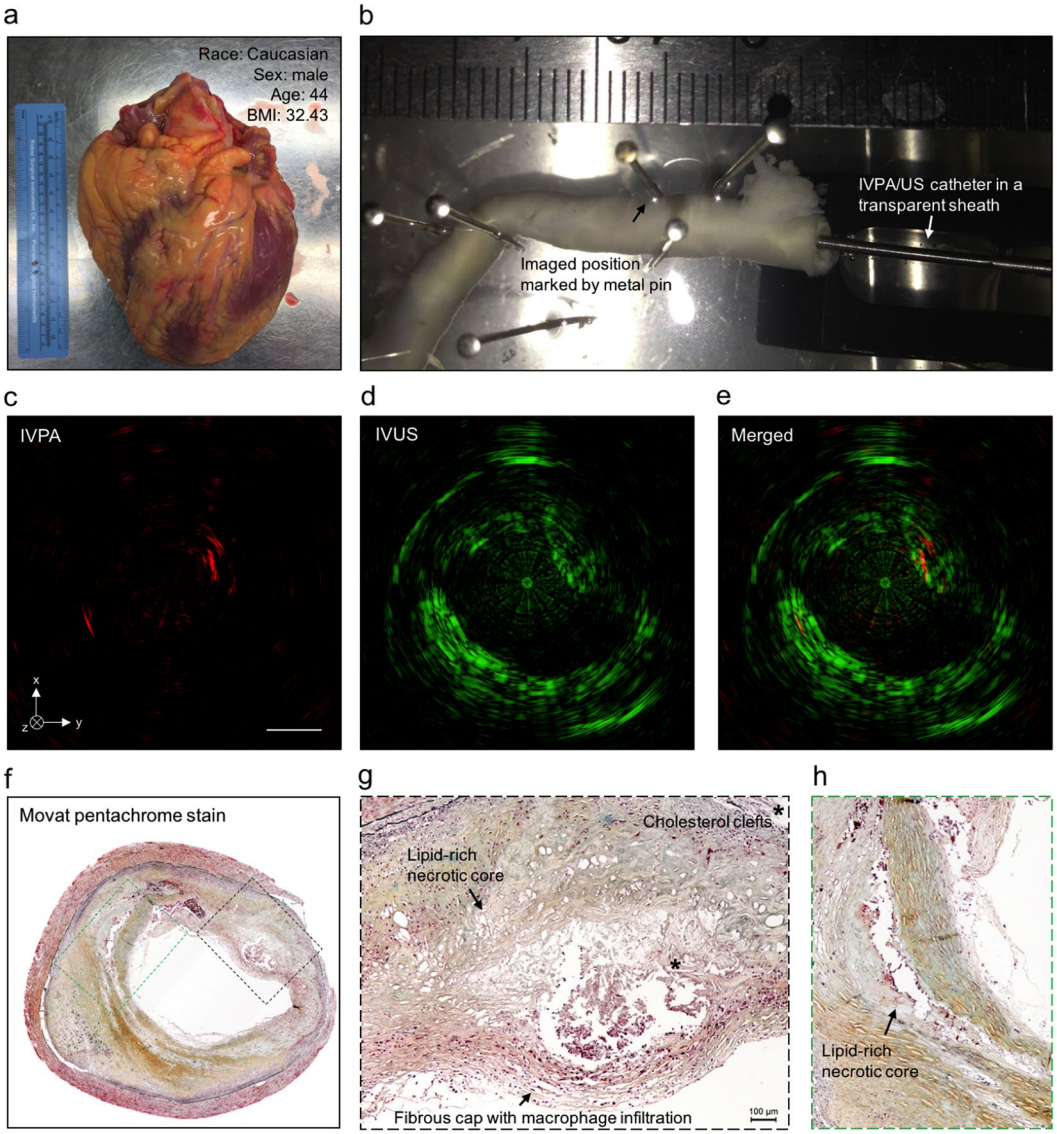
IVPA-US imaging of human coronary atherosclerosis at 16 fps with comparison to histopathology. (**a**) Picture of collected human heart. (**b**) Scenario picture of *ex vivo* IVPA-US imaging of dissected human coronary artery. The region of interest was marked by metal pin. The catheter and sheath were inserted into the artery lumen. Cross-sectional (**c**) IVPA, (**d**) IVUS, and (**e**) merged images of human coronary artery at the region of interest. (**f**) Gold-standard histopathology stained with Movat’s pentachrome at the region of interest. (**g**,**h**) Magnified images of lipid deposition sites corresponding to the dashed boxes in (**f**). *Indicates the accumulation of cholesterol clefts.

To further validate the imaging system for the detection of lipid-laden plaques, a diseased human coronary artery was imaged *ex vivo*. The right coronary artery was dissected from a human heart, collected from a 44-year old male who died of atherosclerotic hypertensive cardiovascular disease (gross image was shown in Fig. 7(a)). The artery sample was pressure-fixed in 10% w/v formalin and immobilized in a Sylgard tray with metal pins and submerged in heavy water as shown in Fig. 7(b). A protective polyimide sheath was used to enclose the IVPA-US catheter except for the imaging window at the catheter tip. The catheter and sheath were inserted to the artery lumen for IVPA-US imaging at 16 fps and with a pulse energy of 80 µJ (40.93 mJ/cm^2^ for laser fluence) on the sample, which is below the 1.0 J/cm^2^ ANSI safety standard for skin exposed in 1.7 µm laser^36^. A positive region of interest was identified based on IVPA, IVUS, and merged images in Fig. 7(c–e), which was further marked with a metal pin as shown in Fig. 7(b). There were two sites of lipid deposition at the 2 and 8 o’clock positions, as recognized by strong signals in the IVPA channel (Fig. 7(c)). There was a noticeable lipid-rich core at the 2 o’clock position, which correlated with luminal encroachment observed in the IVUS channel (Fig. 6(d)). The co-registered IVPA-US image (Fig. 7(e)) suggested that this lipid-rich core was beneath a fibrous cap of the plaque surface shown by signal in the IVUS channel. To validate our imaging results, we performed gold-standard histopathology at the region of interest. Fig. 7(f) provided an overall image of histology. The lumen and artery structure in the histological section correlated with artery morphology as shown in the IVUS channel. Furthermore, the areas between 5 and 9 o’clock was rich in fibrous tissue, correlating with the strong echogenicity observed in the IVUS channel at the same location. Also apparent were two lipid-rich necrotic cores (Fig. 7(g,h)), as identified by the loss of matrix, cholesterol clefts, and macrophage infiltration into the lipid pool with an overlying fibrous cap (200 µm thickness). Based on these histological hallmarks, we identified this plaque as an advanced fibroatheroma, which showed strong correlation with our imaging results.

## Discussion

In summary, we have demonstrated a real-time IVPA-US imaging system for the detection of lipid-laden plaque in a human coronary artery at 16 fps. In this system, we presented several key innovations, including excitation laser source, fiber-optic rotary joint assembly, 1 mm diameter IVPA-US catheter, and real-time image processing and display algorithms. By imaging pulsatile motion at different imaging speeds, 16 fps was determined to be adequate to suppress motion artifacts from cardiac pulsation for future *in vivo* applications. Using this system, we further imaged and identified a lipid-laden plaque *ex vivo* in a human coronary artery, which was confirmed by gold-standard histopathology. Collectively, this work offered significant advances towards the clinical translation of IVPA-US imaging technology.

Imaging speed has been a remaining challenge in development of IVPA/US imaging technology, primarily limited by laser pulse repetition rate. In this work, we overcame this obstacle by using a MOPA-pumped OPO with a 2 kHz optical excitation and achieved up to 25 fps imaging speed, making the IVPA-US system comparable to the commercial IVUS and NIRS-IVUS systems. Although a video-rate speed at 30 fps is preferred for *in vivo* applications, achieving so would potentially be cost-prohibitive during laser sourcing and development. Moreover, our imaging results concluded that 16 fps imaging speed does not suffer from motion artifacts induced by cardiac pulsation. Meanwhile, this speed was achieved without significantly compromising image quality with the reciprocal reduction of number of A-lines per image frame. To further improve the IVPA imaging quality at 16 fps, we can use a number of strategies including slightly increasing the laser repetition rate (but number of A-lines does not necessarily to be the same as that in IVUS channel) or using less A-lines per image frame paired with advanced reconstruction algorithms that exploit speckle differences in PA and US imaging^37^.

Real-time imaging capability is also a critical feature for clinical translation of the IVPA-US technology. It can provide operator the necessary and timely feedback to adjust imaging parameters based on their operational requirements. These include the laser pulse energy, the amplification of signals, the pullback speeds, and the image processing parameters. Moreover, the real-time imaging allows to accurately locate the catheter tip at the time of imaging and instantly revisit an identified region of interest. The high-speed real-time imaging capability, along with the superior chemical selectivity and high scalability of PA imaging, makes the imaging system versatile for other intravascular and endoscopy applications. By using a different excitation wavelength or multiple wavelengths, our system could be used to detect other plaque components, e.g. intraplaque hemorrhage or loss of collagen in the fibrous cap. Furthermore, exogenous contrast agents could be employed to target other markers of plaque vulnerability such as macrophage infiltration or matrix metalloproteinases^38–40^. Our system may also resolve the current limitations (slow imaging speed, 4 Hz; partial field of view) encountered in PA endoscopy of gastrointestinal tracts^41^.

Our system is currently limited by the lack of an optically and acoustically transparent sheath fully enclosing the IVPA-US imaging catheter. A sheath is a necessary component in a clinical setting to protect the catheter components and the artery endothelium. In addition, the sheath must be flexible and have a clinically compatible size. Lastly, a clinically relevant animal model of atherosclerosis will be critical in refining the catheter and the sheath designs and preclinical validation of the technology *in vivo*.

## Methods

### MOPA-pumped OPO

The detailed optical layout of the laser was shown in Fig. 2(a). In the MOPA configuration, a Nd:YAG laser side-pumped by a laser diode was used as the master oscillator, generating 2.5 mJ and 15 ns laser pulses at a frequency of 2 kHz and a wavelength of 1064 nm. The output of the laser diode was collimated onto the Nd:YAG crystal through a pair of coupling lenses. A BBO Pockels cell was used as the electro-optical Q-switch along with a polarizer and a quarter-wave plate. A Nd:YAG rod side-pumped by a collimated laser diode was applied as the power amplifier. After the amplification, the pulse energy reached 6.0 mJ at a wavelength of 1064 nm and a pulse duration of 15 ns. The wavelength of the laser pulses was shifted to 1.7 µm as the final output by a KTP-based OPO. In the OPO cavity, two KTP crystals were specially cut to realize the type II phase matching and placed with opposite orientation to effectively reduce the walk-off effect. The temperature of the laser rods and the KTP crystals were kept at 295 K for effective heat dissipation. All optical components were installed and enclosed in a compatible aluminum alloy box with dimensions of 107 × 260 × 430 mm^3^. The performance of the laser output was further characterized as shown in Fig. 2(b,c) and Supplementary Fig. S1.

### IVPA-US catheter

We designed and assembled a new 1 mm catheter for simultaneous IVPA and IVUS imaging. The detailed design was shown in Fig. 3(a,b). In the catheter, a multimode fiber (FG365LEC, Thorlabs) was used to guide the optical beam. The proximal end of the fiber was polished to 90° and assembled with a FC/PC connector for light coupling. The distal end was polished to 47° to realize the collinear overlap between the optical and acoustic paths (Supplementary Fig. S2), considering the small difference in refraction index between the fiber core (1.44) and water or heavy water (1.33). A single element ultrasound transducer (center frequency, 40 MHz; bandwidth, 52%; size, 0.5 × 0.6 × 0.2 mm^3^; Blatek Inc.) was used to receive generated PA and US signals sequentially. A 45° rod mirror (made from the same fiber, polished to 45° and coated with gold, with an optical reflection of 99% and a damage threshold of 1 J/cm^2^) was used to reflect both optical and acoustic waves. The catheter housing (Proto Labs Inc.) was 3-D printed with micro-resolution stereolithography process to enclose and align the relative positions of fiber distal end, rod mirror, and ultrasound transducer. The fabrication was completed on a workstation capable of aligning these components precisely and monitoring the PA and US signals in real-time under aqueous environment.

### Data acquisition, image processing and display algorithm

In order to process and display IVPA-US images in real-time, we developed LabView-based algorithms and an intuitive graphical user-friendly interface. First, we used a differentiated A-line strategy for IVPA and IVUS imaging by doubling the US triggering frequency (Fig. 4(a)). Specifically, the TTL signal from the laser was used to trigger data acquisition. The same signal was also used to generate two delayed TTL signals (one channel was delayed for 8 µs and the other for 258 µs) through a delay generator (9512+, Quantum Composer). The two channels were further multiplexed for triggering ultrasound pulser/receiver. Thus, the signal recorded in each acquisition cycle contained one PA signal segment, two US signal segments, and the remaining non-meaningful signal. The signal was then processed by developed image processing and display algorithms (the processing flow shown in Supplementary Fig. S3). Finally, the IVPA and IVUS images were displayed separately on screen in real-time. Using the same methodology as that in 16 fps imaging, we achieved 10, 20, and 25 fps real-time image processing and display. In 10 fps IVPA-US imaging, 200 and 400 raw A-lines were used to reconstruct cross-sectional IVPA and IVUS images, respectively. Similarly, for 20 fps, 100 and 200 raw A-lines for IVPA and IVUS images; for 25 fps, 80 and 160 raw A-lines for IVPA and IVUS images. For 1 and 5 fps, the US triggering frequency was the same as PA, thus IVPA and IVUS images had the same number of A-lines. For 1 fps, IVPA and IVUS images both had 2000 raw A-lines. For 5 fps, both images had 400 raw A-lines. For off-line image reconstruction, an additional median filter was used to reduce noise in both IVPA and IVUS images. An A-line interpolation method was further implemented into the reconstruction algorithm smoothing both IVPA and IVUS images. This was a method that applied interpolation for raw data points along the lateral direction, through which the discontinuity between raw A-lines can be smoothed.

### IVPA-US imaging system

The portable IVPA-US imaging system was shown in Fig. 1(a,b). In the system, the laser output was coupled to a multimode fiber (FG365LEC, Thorlabs) through a fiber coupling mount (Supplementary Fig. S4). A designed fiber-optic rotary joint (Fig. 3(c,d)) delivered the laser beam to the IVPA-US catheter tip sequentially through the following optical parts: SMA collimator (F220SMA-C, Thorlabs) for beam collimation, a second SMA collimator (F220SMA-C, Thorlabs) used for coupling the beam into a terminated fiber end, a SMA-to-SMA mating sleeve (ADASMA, Thorlabs) used to properly align the cores of the each terminated fiber end and minimize back reflection. Each terminated fiber end was assembled with a SMA connector. The second collimator, the short fiber segment, and the mating sleeve were fixed in place and enclosed by a metal rotor. Three bearings were assembled onto the proximal end of the rotor and then installed into a stator; while the first collimator was fixed in place into the other end of the stator. The rotator drove the rotor and the optical components in the rotor at a set speed. A step motor (X-NMS17C-E01, Zaber) was used to drive the rotator through a belt for cross-sectional scanning; a linear stage (X-LRM100A, Zaber) was integrated for pullback. The speeds of rotational scanning and linear pullback were controlled by a computer. The optical pulses and initial ultrasound pulses were sequentially sent to the IVPA-US catheter through the multimode fiber and the electric wire of the ultrasound transducer, respectively, to generate IVPA and IVUS signals. The detected signal was transmitted through a slip ring. Electronic noise induced by the assembly was suppressed by electrical grounding and shielding. The signal was received and amplified (39 dB) by an ultrasound receiver (5073PR, Olympus, Inc.), digitized and acquired by a DAQ card (ATS9350 PCI express digitizer, AlazarTech) with waveform digitization of 12-bit, sampling rate of 500 MS/s, and data throughput of 1600 MB/s. The digitized data was processed and displayed in real-time by the computer. The laser chiller used was a non-compressor based chiller. This robust, compact, and portable design is essential for future work in catheterization labs for preclinical and clinical studies.

### Human artery samples and histology

Human specimens were obtained within 24 hours of death from deceased patients bequeathed to Indiana University School of Medicine, as approved by Institutional Review Board exemption. The studied donor sample was from a 44-year old Caucasian male with a body mass index of 32.4, past medical history of a previous myocardial infarction, smoking and alcohol use, and hypertension, and died from atherosclerotic hypertensive cardiovascular disease. The right coronary artery was excised from surrounding bulk myocardium. Small side branches were ligated with ligation clips to allow for pressure-perfusion and the proximal portion of the artery was cannulated using a modified 6 F hemostatic introducer sheath. To maintain the vessel morphology, we pressure-fixed the artery using 10% w/v formalin with a large barrel syringe (140 mL Monoject, Covidien) for 20 minutes at 225 mL/min, which approximately translated to a physiologic pressure. The artery was left in formalin overnight to ensure fixation. We imaged the vessel by first submerging and perfusing it with room temperature 1X PBS, pH 7.4 to remove all air. Next, we inserted the IVPA/US catheter, partially enclosed with a polyimide sheath, through the hemostatic introducer sheath and advanced it distally to a point of resistance (from tortuous anatomy). We completed a pullback of the entire artery length at a pullback rate of 0.2 mm/s and 1 fps imaging speed. After we identified a positive region of interest (an area with strong signal from the IVPA channel), we repeated imaging at 16 fps. We identified and marked the catheter tip location in the vessel at our imaged region of interest by using a metal pin that was strongly echogenic on IVUS. This region was grossly segmented into a ~2.5 mm section and further sectioned at multiple 250 µm levels and stained with Russell-Movat’s pentachrome.

### Ethics statement

All the experiment protocols in this study were approved by the Institutional Biosafety Committee of Purdue University, and in accordance with the approval guidelines. The experiments involving human artery samples were approved by Human Research Protection Program of Purdue University, and the informed consent was obtained from all subjects.

## Electronic supplementary material

Supplementary Information

Supplementary Video S1

Supplementary Video S2

Supplementary Video S3

Supplementary Video S4

Supplementary Video S5
