# Supplementary material for: Real-time intravascular photoacoustic-ultrasound imaging of lipid-laden plaque in human coronary artery at 16 frames per second
Source: Sci Rep. 2017 May 3;7:1417. doi: 10.1038/s41598-017-01649-9 (PMC5431181; doi:10.1038/s41598-017-01649-9)
Supplement: Supplementary file 1 — Supplementary Information [file 41598_2017_1649_MOESM1_ESM.pdf]

Real-time intravascular photoacoustic-ultrasound imaging of lipid-laden plaque in human coronary artery at 16 frames per second

Jie Hui, Yingchun Cao, Yi Zhang, Ayeeshik Kole, Pu Wang, Guangli Yu, Gregory Eakins, Michael Sturek, Weibiao Chen, Ji-Xin Cheng

Supporting figures and captions

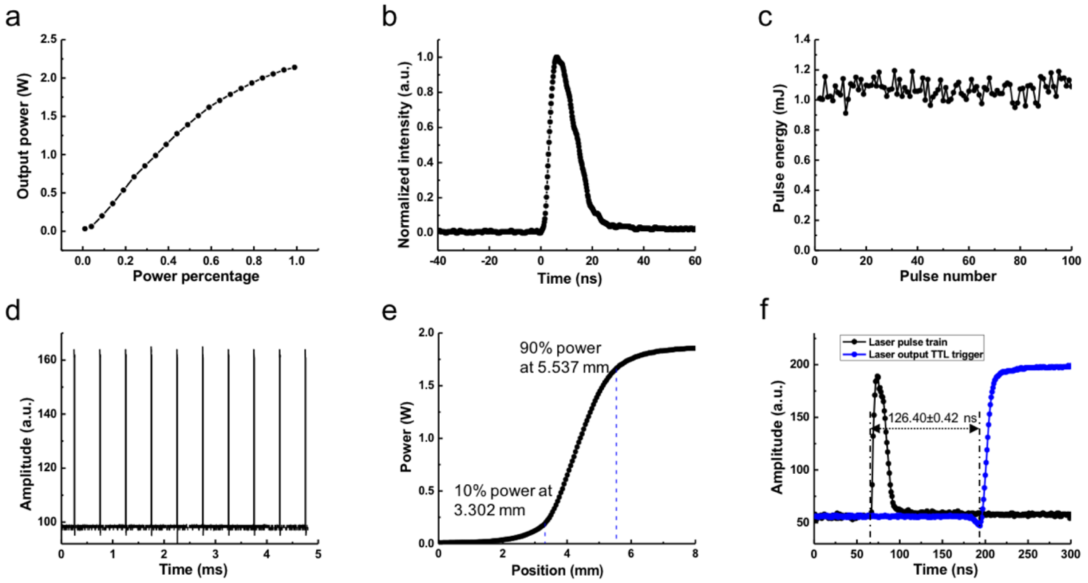

Supplementary Figure S1: Performance of 1.7 μm 2 kHz MOPA-pumped OPO. (a) Output power with power percentage. This was a second way to control the laser output power in the range of 0-2.1 W (0 to 1.05 mJ for pulse energy) by changing the power percentage. The percentage refers to the output power to its maximum power, with the output power controlled by the pump delay of 144 to 27 μs. (b) Pulse width of 11.46 ns$\pm$0.68 ns was measured by the FWHM of single pulse profile measured with an output of 1.5 W. (c) Pulse-to-pulse energy variation was 5.6%,

indicating that pulse energy normalization for each A-line was not necessary. (d) Laser pulse repetition rate, 2 kHz, measured by a fast photodiode. (e) Output beam waist measurement by knife-edge method. The waist was 1.75 mm, a parameter used in laser fiber-optic coupling. (f) Delay between laser pulse and output TTL trigger was 126±0.42 ns, which can be regarded as constant and used for precise event triggering and synchronization.

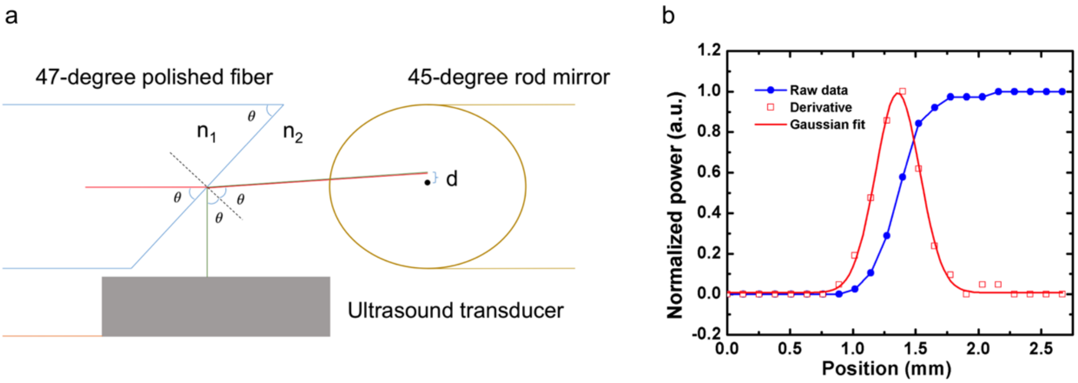

Supplementary Figure S2: Laser beam characteristics at catheter tip. (a) Schematic of optical and acoustic paths in catheter tip with top view. Red line, optical path; green line, acoustic path. In this design, the optical path in the catheter tip was refracted by the silica-water interface, while the acoustic path was reflected by the same interface. Both paths were collinearly overlapped after the interface when the fiber-polishing angle, θ, was 47°. The angle was calculated by equation $n_1 \sin(90 - \theta) = n_2 \sin(\theta)$, where n_1 is 1.44, refraction index of pure silica in fiber core and n_2 is 1.33, refraction index of water or heavy water. The deviation angle of optical path caused by refraction was 4°, a very small angle. As the distance between the center point of the interface to the center point of rod mirror, D, was ~500 μm, the distance between the optical reflection spot to the center point of the rod mirror, d, was calculated as $d \approx D\sin(4°) = 35$ μm. This distance was small relative to the area of reflection surface of the rod mirror, so the laser pulse was effectively

reflected by the rod mirror. The 45° rod mirror was used to reflect the laser pulse to samples and excite samples nearly perpendicularly at catheter output window. (b) Laser output profile at catheter tip characterized by knife-edge measurement. The blue dots indicated the output power measured along different positions of knife edge. The derivative of raw data showed that the output beam had a Gaussian or Gaussian-like profile. The beam waist (ω =0.353 mm) was estimated by Gaussian fitting the derivative points. The laser fluence, ϕ, was calculated by equation $\phi = \frac{E}{\pi\omega^2/2}$, where E is the pulse energy.

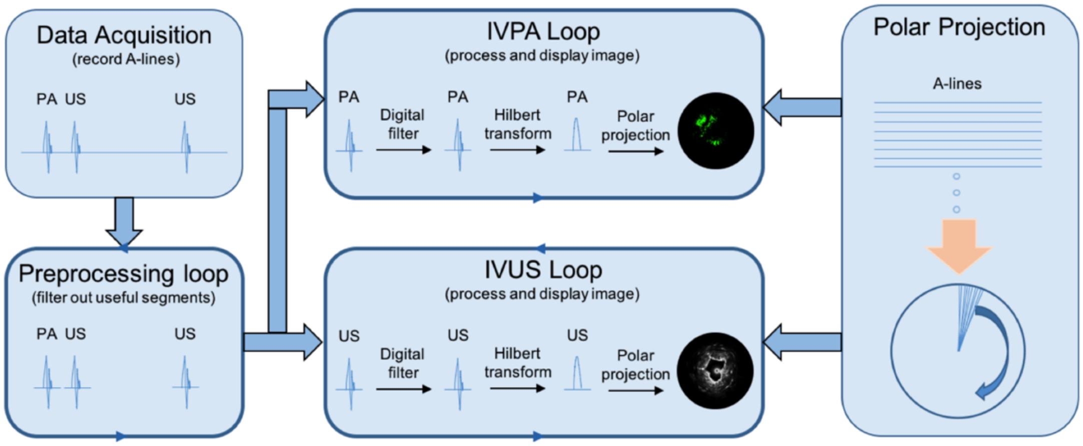

Supplementary Figure S3: Real-time IVPA-US image processing and display. The signal recorded in each cycle contained one PA signal segment, two US signal segments, and the remaining non-meaningful signal. The DAQ card recorded and digitized the signal in each cycle. The remaining signal was discarded in a preprocessing loop and the PA and US signal segments were streamed separately into parallel IVPA and IVUS loops. Both IVPA and IVUS loops processed the signal segment sequentially through the following functions: digital bandpass filter (2 to 50 MHz bandpass); Hilbert transform for signal envelop; polar projection to convert the series of A-line necessary for a cross-sectional image into a polar image. The polar projection module

generated a look-up table, through which Cartesian coordinates were converted into polar coordinates for IVPA and IVUS images simultaneously. This module was initialized before each data acquisition and display, which means that the look-up table was only calculated once before recording and displaying the data.

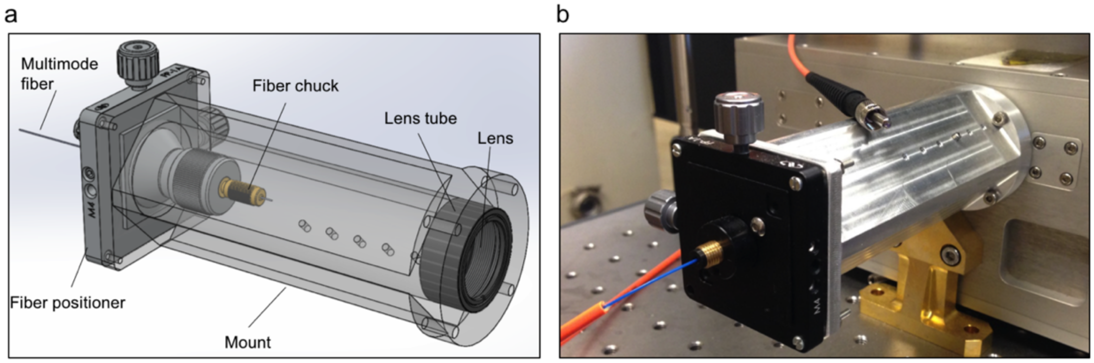

Supplementary Figure S4: Fiber-optic coupling mount for high-power laser. (a) Schematic of the coupling mount design. The fiber was held by a fiber chuck (FPH-DJ, Newport) and its position was precisely tuned for optical coupling by a fiber positioner (FP-1A, Newport). A focal lens (f, 60 mm; LB1596-B, Thorlabs) was used to match the multimode fiber mode field enabling 96% coupling efficiency under high energy and high repetition-rate conditions. It tolerated the maximum output of 2.1 W or possibly even more. (b) Picture of the fiber-optic coupling mount. It was assembled and mounted on the laser box at its output window.

Supplementary Video Captions

Supplementary Video S1: A demonstration video of real-time IVPA-US imaging of dynamic pulsatile motion at 16 fps. The entire imaging system was assembled on a compact and mobile

cart. The IVPA and IVUS image was shown simultaneously on the display monitor. The corresponding IVPA and IVUS A-line signals were also monitored in real-time on an oscilloscope. The fiber-optic rotary joint assembly guided 1.7 μm excitation laser pulse to the IVPA-US catheter tip with a rotation speed of 30 revolutions per second. A sheath was used to protect the IVPA-US catheter under high-speed rotation. A heat shrink tube segment with mechanically generated pulsatile motion was imaged. We acknowledge Rigol, Olympus, Quantum Composers, and NIALT for their permissions for the use of company logos.

Supplementary Video S2: A demonstration video of real-time IVPA-US imaging of dynamic pulsatile motion at 25 fps. Similar with 16 fps imaging, a heat shrink tube segment with mechanically generated pulsatile motion was imaged at 25 fps. The IVPA and IVUS image was shown simultaneously on the display monitor. The corresponding IVPA and IVUS A-line signals were also monitored in real-time on an oscilloscope.

Supplementary Video S3: Merged IVPA-US imaging of heat shrink tube with 1.2 Hz dynamic pulsatile motion at speed of 1, 5, 10, 16, 20, and 25 fps. The video corresponds to Fig. 5. The four consecutive IVPA-US image frames in Figs. 5(a)-(d) were selected as the representative images from the video at 1, 5, 10, and 16 fps to demonstrate image distortion caused by pulsatile motion. The pulsatile motion mimicking a human heartbeat was generated by mechanically pinching the tube at 1.2 Hz with forceps. All the panels in the video displayed cross-sectional IVPA-US images with a total time of 30 s. The red and green colors represent IVPA and IVUS signals, respectively.

Supplementary Video S4: IVPA-US imaging of tissue phantom at 16 fps with a pullback length of 10 mm. The video corresponds to Fig. 6. Figs. 6(b)-(d) were selected as representative images from the video. The pullback speed is 0.5 mm/s, indicating a total 320 image frames within a 20 s display time.

Supplementary Video S5: 3D rendered IVPA-US images of tissue phantom at 16 fps with a pullback length of 10 mm. The video corresponds to Fig. 6. Figs. 6(f)-(h) were selected as representative 3D images from the video. To clearly reflect the 3D tissue phantom structure, the noise in the center of the image was suppressed.
